# Roles of Mir-144-ZFX Pathway in Growth Regulation of Non-Small-Cell Lung Cancer

**DOI:** 10.1371/journal.pone.0074175

**Published:** 2013-09-16

**Authors:** Wangjian Zha, Liu Cao, Ying Shen, Mao Huang

**Affiliations:** 1 Department of Respiratory Medicine, The First Affiliated Hospital, Nanjing Medical University, Nanjing, China; 2 Department of Ophthalmology, Nanjing First Hospital, Nanjing Medical University, Nanjing, China; 3 Division of Neurosurgery, Moores Cancer Center, University of California San Diego, La Jolla, California, United States of America; University of Barcelona, Spain

## Abstract

**Background:**

Lung cancer is the leading cause of cancer-related death worldwide. Non-small cell lung carcinoma (NSCLC) accounts for most of the lung cancer cases and the prognosis of this disease remains poor despite decades of intensive investigation. Thus new insights into underlying mechanisms by which NSCLC develops are avidly needed as the basis for development of new lines of therapeutic strategies. The past decade has witnessed a growing interest on the regulatory roles of micro RNAs on various categories of malignancies. Related data has been well documented in carcinogenesis and pathophysiology of a variety of malignancies. Even so, there is a relative lack of data on roles of mir-144 in tumor biology and there has been no report showing the involvement of mir-144 in NSCLC development.

**Methods/Principal Finding:**

From human NSCLC tumor tissue samples and cell culture samples, we found that the expression of mir-144 is associated with malignant phenotype of NSCLC. Further investigations showed that ectopic mir-144 expression dramatically inhibits NSCLC tumor cell growth and induces apoptosis as manifested by elevated apoptotic protein markers and flowcytometry change. Moreover, we also found that ZFX protein expression is also associated with malignant phenotype of NSCLC and knockdown of ZFX protein results in a similar effect as of ectopic mir-144 expression. Finally, we found that ZFX expression is highly adjustable upon presence of mir-144 and ectopic expression of ZFX dramatically dampens mir-144 action of tumor inhibition.

**Conclusions:**

Our results for the first time showed mir-144-ZFX pathway is involved in the development of NSCLC, which sheds a light for further investigations on underlying mechanisms toward better understanding and management of NSCLC.

## Introduction

Lung cancer is the leading cause of cancer-related death worldwide [1]. Non-small cell lung carcinoma (NSCLC) accounts for about 80% of total lung cancer cases. Also, this disease is notoriously devastating at an advanced stage [2]. Therefore, a better understanding of the molecular mechanisms involved in NSCLC development is avidly needed as the basis to identify novel therapeutic targets and develop new strategies for the treatment of these diseases.

MicroRNAs are ubiquitously expressed small RNAs which exert negative regulatory effects on gene expression at a post-transcriptional level [3], [4]. Given the fact that microRNAs theoretically target any mRNA, it is likely that microRNAs possess a very broad functional spectrum which includes cell cycle regulation, cell growth, apoptosis, cell differentiation and stress response [5]–[9]. Consistent with this notion, it is no surprise that microRNAs are extensively involved in human cancer development. Although many miRNAs have been reported to be involved in etiology and pathogenesis of cancer by targeting oncogenes or tumor suppressors [10]–[12], the studies addressing the roles of miRNAs in cancer development are still at an early stage.

Recently, there is a growing research interest on the role of microRNA-144 in tumorigenesis and cancer treatment. Several research groups have reported down-regulation of mir-144 in different types of cancers including osteosarcoma and mesothelioma that implied mir-144 as a potential tumor suppressor [13], [14]. More specifically, a recent study revealed an inverse correlation between mir-144 level and gastric cancer development [15]. Two recent papers from Courtney's group showed that knockdown DCAMKL-1 can increase microRNA-144 which in turn contributes to inhibition of colorectal cancer and pancreatic cancer [16], [17]. However, there are also contradictive reports. As for colorectal cancer, another group reported an elevated level of mir-144 in human feces and cancer tissue [18]. A report from Fu et al claimed that mir-144 promotes cell proliferation, migration and invasion in nasopharyngeal carcinoma through repression of PTEN. The function of mir-144 in tumorigenesis and cancer development seems to be complicated and highly tissue-specific [19].

To date, to the best of our knowledge, there has been no report paper specifically addressed the role of mir-144 in lung cancer. However there are several papers discerning the important function of mir-451, another microRNA sharing the same locus with mir-144, in the tumorigenesis and development of lung cancer [20]–[23]. Mir-451 has been reported to be downregulated in lung cancer, and in an inverse relationship with disease occurrence and development. Given that clustered miRNA are usually coordinately transcribed, we hypothesize that mir-144 level is also lower in lung cancer [24], [25]. Interestingly, a recent paper reported a down-regulated mir-144 expression in whole blood of patients with lung adenocarcinoma [26]. These data impelled us to hypothesize that mir-144 expression is also down-regulated in lung cancer, and it has an inhibitory function on proliferation and metastasis of lung cancer cells.

Zinc finger X-chromosomal protein (ZFX) belongs to ZFY protein family [18] and is one of a few genes on the human X chromosome that are known to escape X inactivation. Previous research showed that ZFX has an important role in self-renewal and maintenance of both embryonic stem cells and hematopoietic stem cells [27]–[30]. A few reports showed that ZFX serves as a target for mir-144 and exerts regulatory effects on tumor growth [31], [32]. However, there is still lack of data on the role of ZFX on lung cancer behavior and mir-144-ZFX pathway has never been reported in context of NSCLC.

## Materials and Methods

### Reagents and antibodies

Micro-RNA expression kits for mir451 (001105), mir144 (002676) and RUN48 (001006) were purchased from Applied Biosystems. Cytochrome c antibody (6H2) was obtained from Santa Cruz Biotechnology. Anti-ZFX antibody (ab85483), anti-Caspase 3 antibody (ab44976) and anti-GAPDH antibody (ab9485) were purchased from Abcam. Trizol Reagents was purchased from Invitrogen. TaqMan Gene Expression Assay Kits were purchased from Applied Biosystems (Hs01017881_m1 for ZFX and 4308313 for GAPDH). Other reagents include Lipofectamine 2000 reagent (Invitrogen), SuperSignal Substrate Western blotting detection system (Pierce, USA), Guava Nexin Reagent (Millipore). RPMI 1640 medium and fetal bovine serum were purchased from Shanghai Haoran Biological Technology Co. Luciferase Assay Kit and pMIR-REPORT System were purchased from Applied Biosystems. β-Gal Assay Kit was purchased from Invitrogen (Catalog no. K1455-01). Human ZFX-ELISA kit (CSB-EL026467HU) was purchased from CUSABIO BIOTECH CO., Ltd., China. All other chemicals were purchased from commercial sources at the highest purity available.

### Cell culture

A549, CRL-5875 and HTB-183 cell lines were purchased from ATCC (American Type Culture Collection) through the agency by Beijing Zhongyuan Limited, Beijing, China. The 16HBE14o^−^ cell line (human normal bronchial epithelial cell) is a generous gift from Dr Dieter Gruenert (University of California, San Francisco) [33]. All cells were cultured in RPMI 1640 supplemented with fetal bovine serum (10%), 1% nonessential amino acids and penicillin-streptomycin were added. Cells were cultured in a humidified chamber at 37°C with 5% CO_2_.

### Ethics statement

All experimental procedures were approved by the Institutional Review Board of Jiangsu Province Medical Association. The information was given to the patients, and written consent was obtained for all patient samples.

### Plasmid construction

To construct pre-mir-144, a DNA fragment containing the 86-bp hsa-miR-144 precursor (plus 100 bp upstream and 100 bp downstream) was amplified from genomic DNA of 16HBE14o- cells and cloned into pcDNA(+)3.1(Invitrogen) which is modified for puromycin resistance. To ectopically express ZFX, the DNA fragment coding sequence of ZFX was multiplied by RT-PCR and cloned into pBABE-neo vector.

For the luciferase assay, pMIR-REPORT System (Applied Biosystems) was used. The plasmids (pMIR-REPORT-Luciferase-ZFX-3′-UTR and its mutant) were constructed by following methods. The 3′-UTR of ZFX was amplified by RT-PCR. The primers for PCR are: gcgcaagcttcaatacttctacagaacg and gcgcgagctccctatatgcaccagtgac. The amplified product was subcloned into pMIR-REPORT-Luciferase vector between HindIII and SacI sites. QuikChange Site-Directed Mutagenesis Kit (Stratagene) was used to generate pMIR-REPORT-Luciferase-ZFX-3′-UTR-mutant plasmid by using following primers: 5′-cgtgtataGCAGgtttgcct-3′ and 5′-aggcaaacCTGCtatacacg-3′. A plasmid coding β-galactosidase (pMIR-REPORT β-gal control) was used to normalize variability due to differences in cell viability and transfection efficiency.

To knockdown ZFX expression, ZFX shRNA plasmid was constructed. The DNA fragments were synthesized, annealed and inserted into pLKO.1 vector. The mature sense sequence is: 5′-atgtacctgtgtgtattgct-3′.

### Retrovirus and Lentivirus Infections

Retrovirus production was performed as described previously [34] using AmphoPhoenix cells. Lentivirus was packaged using the ViraPower Kit from Invitrogen following the manufacturer's instructions. Virus was applied on the target cells for 24 hours. After infection, cells were exposed to either puromycin (1 µg/ml) or neomycin selection (500 µg/ml).

### Mir-144 over-expression

The pre-mir-144 or its corresponding vector plasmid was transfected into A549 cells with lipofectamine 2000 (Invitrogen). The transfected cells were started for selection by puromycin (1 µg/ml) 24 hours after transfection for 2 days.

### Tissue samples

26 patients who were diagnosed as NSCLC in the Department of Respiratory Medicine of our hospital were included in this study. Before the surgery, none of these patients received any treatment. Tumors and surrounding non-tumor lung tissue samples were collected and stored at −80°C. Before sample collection, ethical approval was obtained from the hospital and informed consent was obtained from all subjects before tissue collection started.

### RNA extraction from tissues and cells

Clean the homogenizer probe, forceps, spatulas and any other instruments which are used to handle the tissue samples by washing them with following buffers sequentially: RNAseZAP, 75% Ethanol and DEPC Water. Allow frozen tissue samples to thaw enough, and then cut the tissue into small pieces with a sterile razor blade. Scrape all the tissue pieces into a 50 ml tube containing buffer L3 (Invitrogen), and homogenize tissue 3 times (30s every time, put samples on ice for the intervals). Spin homogenized samples at 12000 g for 5 minutes at 4°C. Total miRNA was extracted using PureLink miRNA Isolation Kit (Invitrogen) by following the manufactory manual.

To extract RNA from cultured cells, **s**crape cells (about 2×10^6^ for each sample) into 15-ml tubes, and spin them at 250×g for 5 minutes to pellet cells. Add 300 µl buffer L3 to each cell pellet, and vortex. MicroRNA was extracted following the manufactory manual (PureLink miRNA Isolation Kit, Invitrogen).

### Real-Time PCR analyses of miRNAs

The levels of miRNA were determined using TaqMan MicroRNA Assays Kit (Applied Biosystems). Primers for miR-451, mir144 and RUN48 were purchased from ABI. The reverse transcription reaction and real-time PCR were performed according provider's protocol. RUN48 was used as the internal standard for normalization. Relative expression of miRNA was calculated by using average of the control group as a calibrator.

### Real-Time PCR analyses of ZFX mRNA

Total RNA was extracted from cells of different groups using Trizol reagents. The mRNA levels of ZFX were tested using TaqMan gene expression assay kits. Real-time PCR was performed following the manufactory protocol. Data were normalized with the housekeeping GAPDH gene.

### Guava Nexin Assay

The assay was performed following manufactory protocol (Millipore). Briefly, attached and suspended cells were all collected. Cells were suspended in 100 µL of medium and incubated with 100 µL of Guava Nexin Reagent in each well for 20 minutes at room temperature in the dark. Samples then were measured on a Guava System (Millipore). The data were analyzed by using the software provided by the company.

### Western blot

Whole cell lysates were harvested in 2% SDS supplemented with proteinase inhibitors. Equal amounts of proteins (50 µg) were subjected to electrophoresis on a polyacrylamide gel (10 or 15%). Proteins were transferred onto pure nitrocellulose membranes. After protein transfer, membranes were blocked with 5% fat free dry milk in TBS-T for 1 h before addition of primary antibodies and incubated at 4°C overnight. The membranes were then washed three times with TBS-T and incubated with secondary antibodies for 1 h at room temperature in TBS-T. After three washes in TBS-T, immunoreactive products were visualized using the SuperSignal Substrate Western blotting detection system (Pierce, USA).

### Luciferase assay

The luciferase was performed on A549 cells by using pMIR-REPORT System [34] (Applied Biosystems) following the provider's protocol. Briefly, pMIR-REPORT-Luciferase-ZFX-3′-UTR or its mutant (3 µg) was co-transfected with pMIR-REPORT β-gal control (1 µg) into A549 cells (10^6^) with or without mir-144 over-expression using 15 µl of Lipofectmin 2000 (Invitrogen).

24 hours later, the luciferase activity was measured with Luciferase Assay Kit (Applied Biosystems). Rinse cells with PBS. 250 µL Lysis Solution was added to the cells. Detach cells from plate with a cell scraper. Transfer the cell lysate to a microfuge tube and centrifuge at 4°C for 5 min to pellet debris. Transfer supernatant to a fresh tube. Transfer 50 µL of cell extract to a luminometer tube. Add 100 µL of Substrate A (ATP solution). Add 100 µL of Substrate B (luciferin solution). Program the luminometer to perform a 2-s pre-measurement delay followed by a 10-s measurement period. Place the tube in the luminometer and initiate reading. The β-galactosidase activity was tested by using β-Gal Assay Kit (Invitrogen) following the manufacturer's manual. The relative luciferase activity was obtained by normalizing luciferase expression with β-gal expression.

### ELISA assay of ZFX

Thaw samples from −80°C. Cut normal and tumor tissues into small pieces, and weigh about 100 mg of samples for each assay. Rinse tissue with PBS, homogenize tissue in 1 mL of PBS and store overnight at −20°C. Two freeze-thaw cycles were performed to break the cell membranes, centrifuge the homogenates for 5 minutes at 10000 g, 4°C. Measure protein level of ZFX in the supernate by using ZFX-Elisa kit (CUSABIO BIOTECH CO., Ltd., China) following the manufacturer's protocol, and normalize it against the weight of tissue.

### Statistics

Experimental results are shown as the mean ± S.D. Statistical analyses were performed by unpaired Students *t* test or ANOVA assuming unequal variance unless otherwise indicated using SigmaPlot 11.0 (San Jose, CA USA). Significance was defined as *p<0.05, ** p<0.01, *** p<0.001.

## Results

### Levels of mir-144 and mir-451 are decreased in NSCLC tumor tissues comparing to peri-tumor normal tissues

**Figure 1:**
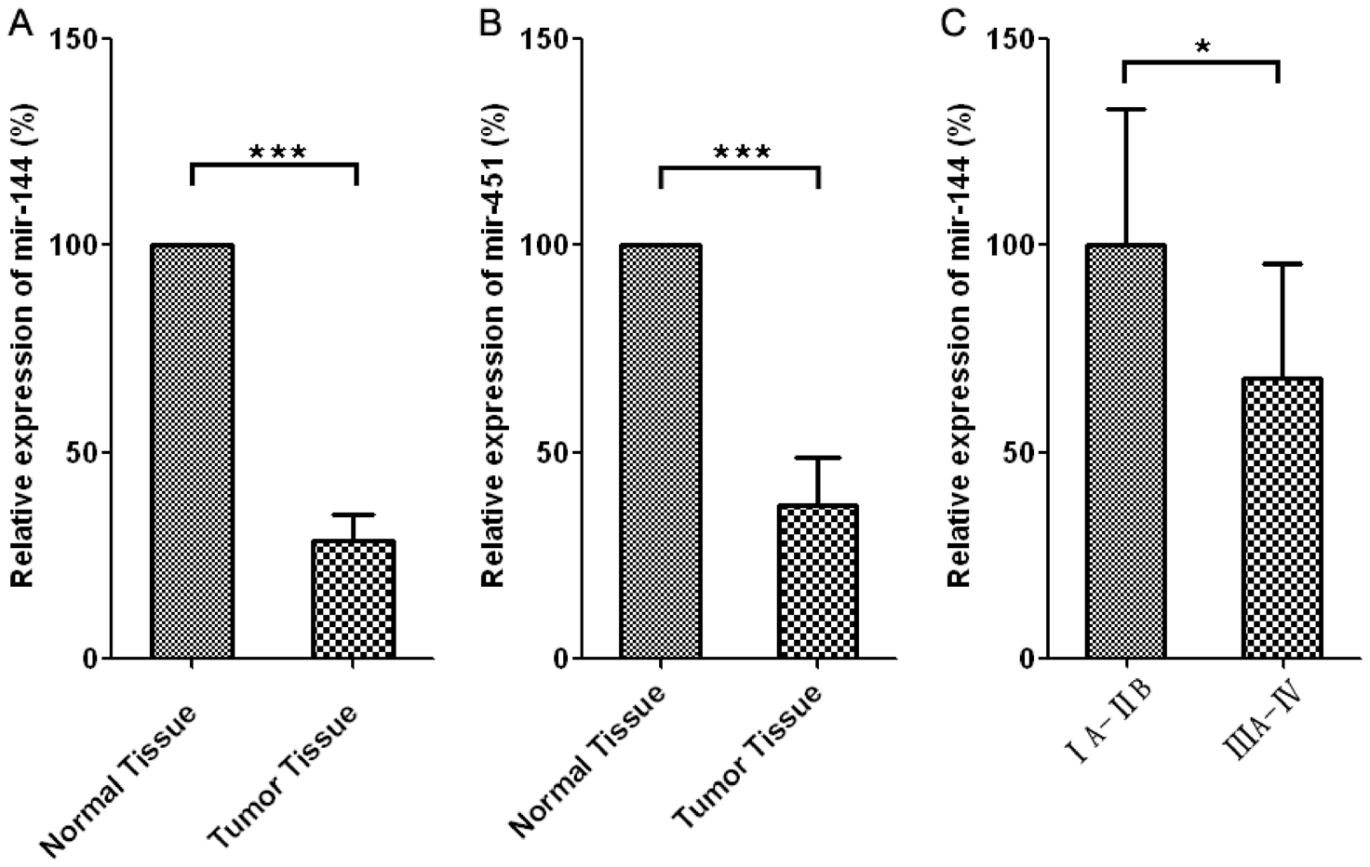
Levels of mir-144 and mir-451 are decreased NSCLC tumor tissues comparing to peri-tumor normal tissues. A and B. The relative expression levels of mir-144 and mir-451 in normal (n = 26) and tumor tissues (n = 26). Expression levels of mir-144 or mir-451 in tumor tissues were normalized to the expression of above microRNAs in the corresponding normal tissues from the same patient. C. The relative expression levels of mir-144 in low-grade (IA-IIB) (n = 14) and high-grade (IIIA-IV) lung adenocarcinoma (n = 12).

As shown in Figure 1A and 1B, we found a significant down-regulation for both mir-144 (>70% decrease, p<0.001) and mir-451(>60% decrease, p<0.001) in non-small-cell lung cancer tissues (n = 26) as compared to the peri-tumoral normal tissues (n = 26), which is in line with previous data showing that mir-144 and mir-451 share the same DNA locus, coordinately transcribed and plays similar roles in various pathophysiological scenarios [13], [24], [35], [36]. In addition, a significant decrease of mir-144 level (Figure 1C) is also observed in high-grade cancer (IIIA-IV) comparing to that in low-grade cancer (IA-IIB).

### Expression levels of miR-144 are lower in human lung cancer cell lines when compared to the normal human bronchial epithelial cells

**Figure 2:**
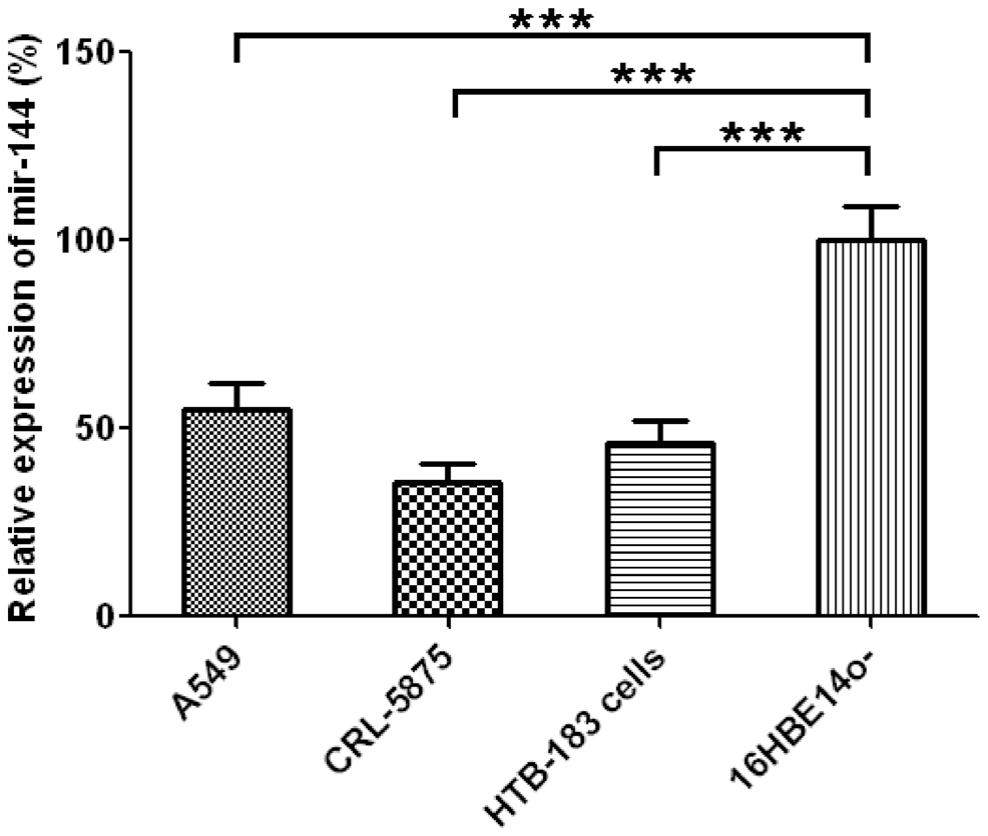
Expression levels of mir-144 in A549, CRL-5875, HTB-183 and 16HBE14o- cells, mean±SD, n = 5.

Consistently, we also found that mir-144 is significantly down-regulated in NSCLC cell lines A549 (>45% decrease, p<0.001), CRL-5875(>60% decrease, p<0.001), HTB-183 (>50% decrease, p<0.001) as compared to normal bronchial epithelial cell lines (16HBE14o- cells) (Figure 2).

### Ectopic miR-144 expression inhibits growth of A549 cells and promotes apoptosis of the cells

**Figure 3:**
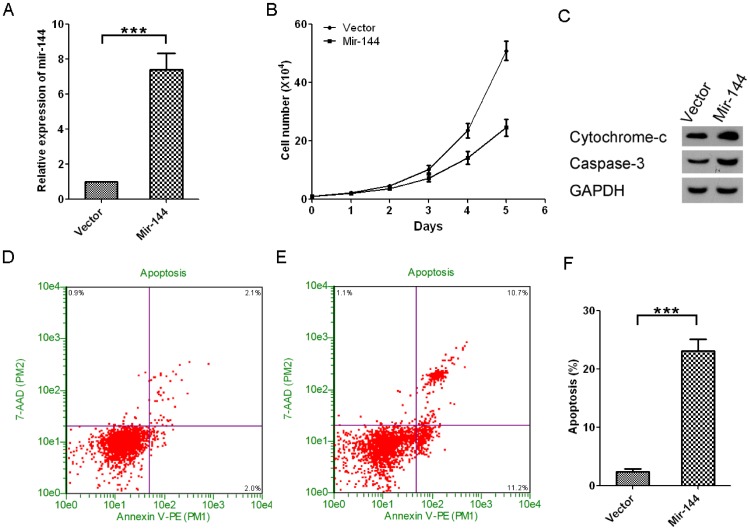
Ectopic miR-144 expression inhibits growth of A549 cells and promotes apoptosis of the cells. A. Over-expression of mir-144 in A549 cells, mean±SD, n = 3. B. Growth curves of A549 cells with or without mir-144 over-expression, mean±SD, n = 3. C. Western-blots of A549 cells with or without mir-144 over-expression. D and E. The representative pictures of Guava Nexin Assay results of A549 cells without and with mir-144 over-expression, respectively. F: Quantification of Guava Nexin Assay results, mean±SD, n = 3.

Next, we sought to determine whether mir-144 plays a direct regulatory role on tumor growth. To test this hypothesis, we successfully engineered mir-144 hyperexpression in A549 lung cancer cells. Results showed mir-144 expression in mir-144 hyperexpressed A549 cell is over 7 fold higher than the vector control (Figure 3A). Interestingly, we found that mir-144 hyperexpression results in significant inhibition of tumor cell growth (Figure 3B) and enhancement of apoptosis as manifested by elevated apoptotic protein markers (cytochrome-c and caspase 3) and flow cytometry results (Figure 3C-F).

The results showed in Figure 1–3 strongly suggested that mir-144 may play an important inhibitory role in lung cancer development. Specifically, it seems that mir-144 is negatively associated with malignant phenotype of lung cancers (Figure 1, 2). In line with this notion, ectopic expression of mir-144 results in a dramatic inhibition of tumor cell growth and an induction of tumor apoptosis.

### ZFX protein, a down-stream target of mir-144, is expressed higher in human lung cancer cell lines than in the bronchial epithelial cell line

**Figure 4:**
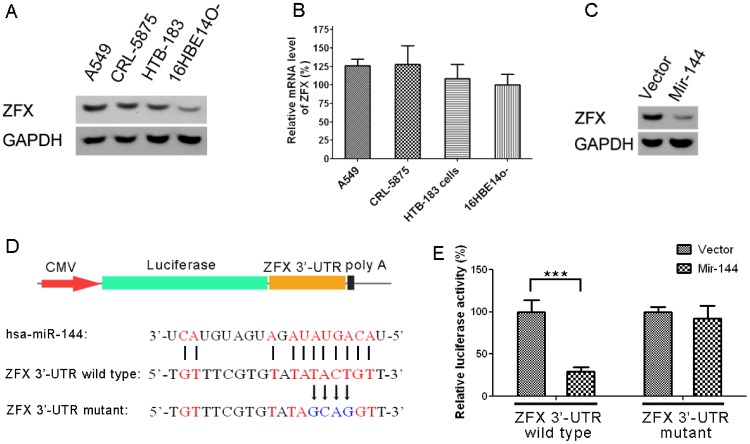
ZFX mRNA and protein expression in the human bronchial epithelial cell line and lung cancer cell lines. The effect of ectopic miR-144 expression on ZFX protein level in A549 cells. A. Relative mRNA expression levels of ZFX in A549, CRL-5875, HTB-183 and 16HBE14o- cells, mean±SD, n = 3. B. Western-blots of ZFX and GAPDH in above cells. C. Western-blots of ZFX and GAPDH in A549 cells with or without mir-144 over-expression. D. The scheme of the luciferase assay using pMIR-REPORT System to evaluate the direct inhibition of mir-144 on ZFX protein expression. E. The different effects of mir-144 on ZFX 3′-UTR and its mutant, mean±SD, n = 5.Mir-144 can efficiently inhibit the luciferase expression by binding to ZFX 3′-UTR, but has no effect on the luciferase expression when the binding sites were mutated on ZFX 3′-UTR.

As the next step, it seems to be intriguing to find out the underlying mechanism for mir-144 mediated tumor inhibition. By comprehensively searching related data, we found ZFX (Zinc Finger Protein, X-linked) may be a potential effector molecule for mir-144 action in context of NSCLC growth and development. To test this hypothesis, we checked protein expression and the mRNA in NSCLC cells and in normal counterparts. As shown in Figure 4A, we found that ZFX protein is significantly higher in NSCLC cell lines as compared to their normal counterpart. We didn't observe a difference between NSCLC and normal cells on mRNA levels of ZFX (Figure 4B). We also found ZFX protein level was lower in mir-144 over-expressed A549 cells when compared to vector control transduced cells (Figure 4C). In Figure 4D we showed the scheme of the luciferase assay using pMIR-REPORT System to evaluate the direct inhibition of mir-144 on ZFX protein expression. We found Mir-144 can efficiently inhibit the luciferase expression by binding to ZFX 3′-UTR, but has no effect on the luciferase expression when the binding sites were mutated on ZFX 3′-UTR (Figure 4E).

### ZFX knockdown inhibits growth of A549 cells and promotes apoptosis of cells

**Figure 5:**
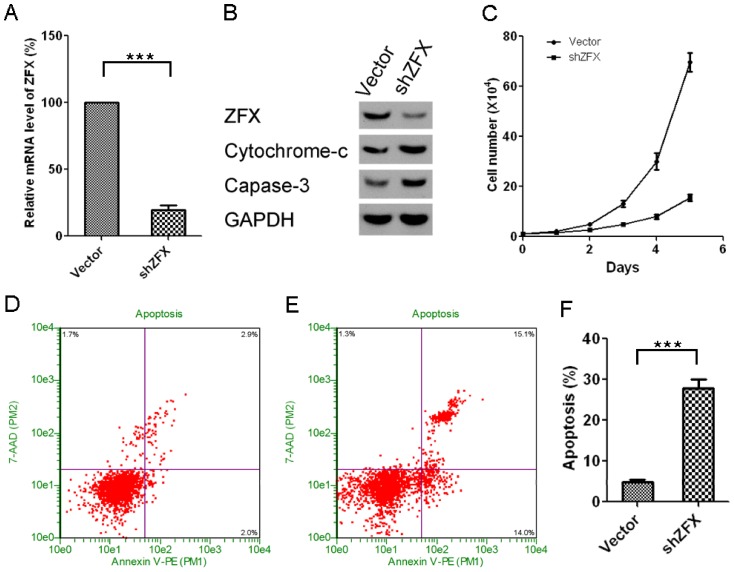
ZFX knockdown inhibits growth of A549 cells and promotes apoptosis of cells. A. Relative mRNA expression levels of ZFX in A549 with or without ZFX knockdown, mean±SD, n = 3. B. Western-blots of ZFX, cytochrome-c, caspase-3 and GAPDH in above cells. C. The growth curves of A549 cells with or without ZFX knockdown, mean±SD, n = 3. D and E. The representative pictures of Guava Nexin Assay results of A549 cells without and with ZFX knockdown, respectively. F: Quantification of Guava Nexin Assay results, mean±SD, n = 3.

To test whether ZFX also exerts direct regulatory function on NSCLC tumor growth, we knocked down ZFX protein in A549 cells. As shown in Figure 5A, our knockdown construct results in >80% of protein expression down regulation as compared to control cells (Figure 5A). As expected, we found that ZFX knockdown strongly induces NSCLC apoptosis (as manifested by enhanced apoptotic protein markers and flowcytometry) and suppresses tumor cell growth (Figure 5B-F), which is similar to the biological effects we observed for mir-144 over-expression shown in previous figures.

### ZFX is required for the suppressive function of mir-144 on NSCLC growth

**Figure 6:**
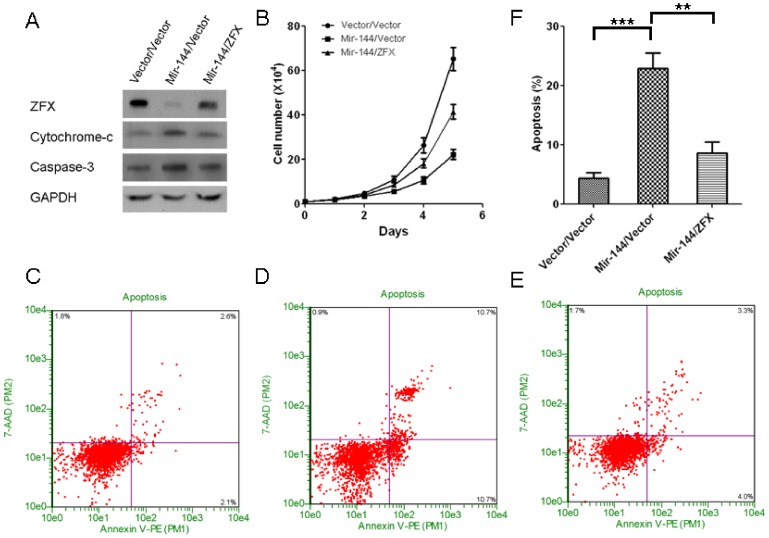
Ectopic ZFX expression rescues growth inhibitory effect of ectopic miR-144 expression in A549 cells. A. Western-blots of ZFX, cytochrome-c, caspase-3 and GAPDH in A549 cells with only mir-144 over-expression, or mir-144 over-expression combined with ZFX over-expression. The control group was transducted with two empty vector coding viruses. B. The growth curves of above cells. C, D and E. The representative pictures of Guava Nexin Assay results of above cells. F. Quantification of Guava Nexin Assay results, mean±SD, n = 3.

To further determine whether ZFX is required for mir-144 action in NSCLC scenario, we simultaneously over-expressed mir-144 and ZFX protein in A549 cell line. We over-expressed mir-144 by transfecting the cells with pre-mir-144 plasmid using lipofectamine 2000. 24 hours later, we infected the cells with retrovirus which coding ZFX protein. Results revealed that ZFX over-expression partially but significantly dampens mir-144 action as demonstrated by decreased cell apoptosis (Figure 6A,6C-6F) and increased tumor cell growth (Figure 6B) compared to the tumor cells with only mir-144 hyper-expression, which clearly suggests that ZFX-depression is required for mir-144 mediated A549 NSCLC cell apoptosis and growth inhibition. Combined with the data shown in Figure 4 and Figure 5, it seems that ZFX as a downstream effector gene, may be involved in mir-144 mediated NSCLC tumor development regulation,which sheds light on further investigation of this novel pathway in NSCLC management.

### ZFX expression levels are significantly higher in tumors when compared to their adjacent normal tissues

**Figure 7:**
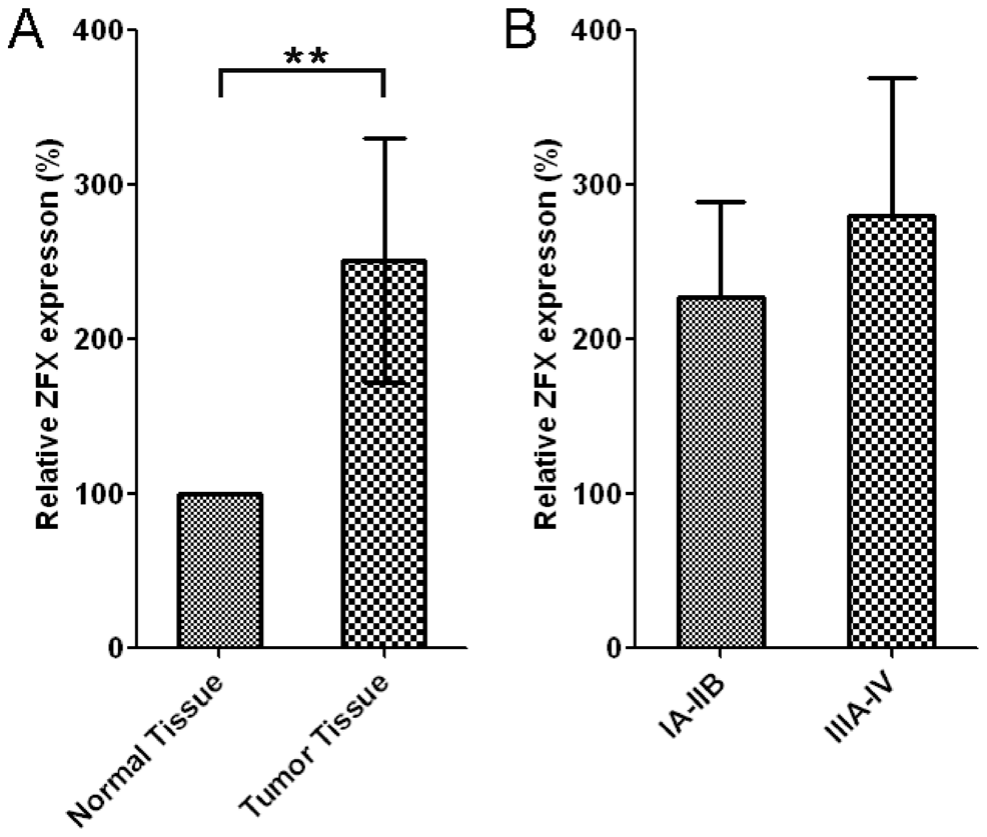
ZFX expression levels are significantly higher in tumors when compared to their adjacent normal tissues. A. The relative expression levels of ZFX in normal (n = 26) and tumor tissues (n = 26). The relative levels of ZFX in tumors were obtained by normalizing against the expression of ZFX in the corresponding normal tissues from the same patient. B. The relative expression levels of ZFX in low-grade (IA-IIB) (n = 14) and high-grade (IIIA-IV) lung adenocarcinoma (n = 12).

Consistent with the previous data, ELISA showed the relative expression levels of ZFX in normal (n = 26) were significantly lower than that in tumor tissues (n = 26, p<0.001) (Figure 7A). The relative levels of ZFX in tumors were obtained by normalizing against the expression of ZFX in the corresponding normal tissues from the same patient. Also, the relative expression levels of ZFX in low-grade (IA-IIB) and high-grade (IIIA-IV) lung adenocarcinoma were also compared. Although ZFX protein levels in high-grade lung agenocarcinoma were mildly higher than those in low-grade, the data was not significant, possiblely because of the small sample size (Figure 7B).

## Discussion

Lung cancer is the most prevalent malignancy worldwide and ranks No. 1 for all cancer death. The current model for lung cancer pathogenesis is believed to be due to the combination of genetic risk factors and untoward environmental stimulations [37]. As discussed before, Non-Small-Cell Lung Cancer (NSCLC) accounts for approximately 80% among all lung cancer subtypes [2]. Therefore elucidation of the underlying mechanism for NSCLC development stands as a major challenge for successful management of lung cancer.

Unfortunately, although the carcinogenesis and pathophysiology of NSCLC have been intensively investigated during the past several decades, the underlying mechanism of NSCLC development remains poorly understood and there is still lack of optimal therapeutic strategies even after decades of intensive investigation.

The past decade witnessed microRNA as a hot area for cancer biology. Although microRNAs are also essential for normal human physiology, many microRNA species have been shown to play important regulatory roles in tumorigenesis and cancer development as well. Examples include, but not limited to, mir-574-3p and prostate cancer[38], mir-23a and gastric cancer[39], mir-21 and colon cancer [40]. Based upon the abundant data accumulated during the past two decades, scientists began to argue for use of microRNAs as novel therapeutic targets for various malignancies[41]. Identified lung cancer related microRNAs include mir-210[42], mir-365[43], mir-449c[44], etc.

In the current study, we were endeavored to provide a new insight into NSCLC development from a perspective of translational science. First, we collected data from bed side, which shows a strongly association between malignant phenotype of NSCLC and mir-144 expression. In light of the data from real NSCLC samples, we further explored the role of mir-144 in NSCLC development. Results turned out to be very positive. Data showed that mir-144 robustly induces apoptosis and inhibits growth of NSCLC cells. Moreover, the results also suggested that the Zinc finger X-chromosomal protein, or ZFX, seems to be a downstream effector molecule for mir-144 action. In terms of NSCLC, it seems that the anti-tumor activity of mir-144 is at least partially through turning down ZFX protein expression at a post-transcription level. This hypothesis was further confirmed with another two key observations 1) Mir-144 exerts direct regulatory roles on ZFX expression and 2) ZFX protein expression (ELISA) seems to be associated with tumorigenesis.

To date, to the best of our knowledge, there has been no report showing a direct involvement of mir-144-ZFX axis in NSCLC development, which warrants further investigation of this pathway in NSCLC behavior. Although we should not rule out the involvement of other target genes for mir-144 action in the current scenario, our results, to a lesser extent, at least provide a proof of principle showing that translational approaches may be useful for future development of novel therapeutic strategies for NSCLC.
